# Where is the evidence for emergency planning: a scoping review

**DOI:** 10.1186/1471-2458-12-542

**Published:** 2012-07-23

**Authors:** Kirsty Challen, Andrew CK Lee, Andrew Booth, Paolo Gardois, Helen Buckley Woods, Steve W Goodacre

**Affiliations:** 1 ScHARR, Regent Court, Sheffield, S1 4DA, UK

**Keywords:** Emergency planning, Disaster, Major incident

## Abstract

**Background:**

Recent terrorist attacks and natural disasters have led to an increased awareness of the importance of emergency planning. However, the extent to which emergency planners can access or use evidence remains unclear. The aim of this study was to identify, analyse and assess the location, source and quality of emergency planning publications in the academic and UK grey literature.

**Methods:**

We conducted a scoping review, using as data sources for academic literature Embase, Medline, Medline in Process, Psychinfo, Biosis, Science Citation Index, Cinahl, Cochrane library and Clinicaltrials.gov. For grey literature identification we used databases at the Health Protection Agency, NHS Evidence, British Association of Immediate Care Schemes, Emergency Planning College and the Health and Safety Executive, and the websites of UK Department of Health Emergency Planning Division and UK Resilience.

Aggregative synthesis was used to analyse papers and documents against a framework based on a modified FEMA Emergency Planning cycle.

**Results:**

Of 2736 titles identified from the academic literature, 1603 were relevant. 45% were from North America, 27% were commentaries or editorials and 22% were event reports.

Of 192 documents from the grey literature, 97 were relevant. 76% of these were event reports.

The majority of documents addressed emergency planning and response. Very few documents related to hazard analysis, mitigation or capability assessment.

**Conclusions:**

Although a large body of literature exists, its validity and generalisability is unclear There is little evidence that this potential evidence base has been exploited through synthesis to inform policy and practice. The type and structure of evidence that would be of most value of emergency planners and policymakers has yet to be identified.

## Background

An effective and efficient emergency response can reduce avoidable mortality and morbidity after a mass casualty incident. Numberous guidance documents have been issued since the Civil Contingencies Act placed a legal responsibility for emergency planning with NHS organisations [1-3]. Following the 7^th^ July 2005 London bombings Lady Justice Hallett’s Rule 43 report raised concerns about the capacity of the London Ambulance service to plan communications for a major incident, the ability to establish a Gold Control Room and the process of triage [4]. Similar comments about the mismatch between plans and events followed the 9/11 World Trade Center attacks [5].

The process for developing policy and guidance documents is variable with the strength and quality of the underlying evidence base often proving unclear. For example the UK Emergency Preparedness document gives no information on the provenance of its evidence base[6]. The review team was commissioned by the UK National Institute of Health Research to conduct a scoping review of the academic and UK grey literature to identify the location, source and quality of emergency planning publications. This would allow us to characterise the evidence that exists to inform emergency planning.

## Methods

### Academic literature identification

Following compilation by the authors of themes and topics considered relevant to the field, pilot searching was carried out in the subarea of health services business continuity. This is an accepted stage for all scoping reviews as it seeks to establish an optimal balance between sensitivity and specificity. This is important conceptually, to finalise the scope of the review, and pragmatically, to ensure that the review is feasible within the available time and resources [7]. A final search strategy was then developed to retrieve evidence relevant to the whole subject area, subdivided into Business continuity, Hazard analysis, Capability assessment and maintenance, Recovery, Communications/informatics and Organisational behaviour/Human Resources. This final search aimed to identify slices of the evidence, but was designed to be more focussed, producing a higher yield of relevant papers and was therefore more time effective to review. The information specialist searched the electronic databases Embase, Medline, Medline in Process and Psychinfo (via Ovid SP), Biosis and Science Citation Index (via Web of Science), Cinahl (via EBSCO), the Cochrane library (via Wiley) and Clinicaltrials.gov (Table 1). Searches were conducted in November 2010 with searches covering the period January 1990 to October 2010. We did not limit to “human” as we wished to identify literature on bioterrorism and zoonoses that might be relevant.

**Table 1:** Search strategies

Business continuity	1. Disasters/pc
	2. (emergency response or emergency preparedness or emergency plan$ or emergency operation plan$ or disaster or major incident$ or incident plan$).ti,ab.
	3. 1 or 2
	4. (business continuity or organisational resilience or business interruption or adaptive capacity or strategic or coordination).ti,ab.
	5. 3 and 4
	6. limit 5 to yr = "1990 -Current"
Hazard analysis	1. Disasters/pc
	2. (emergency response or emergency preparedness or emergency plan$ or emergency operation plan$ or disaster or major incident$ or incident plan$).ti,ab.
	3. 1 or 2
	4. (hazard analysis or risk factor or risk assessment or forecasting simulation or modelling).ti,ab.
	5. 3 and 4
	6. limit 5 to yr = "1990 -Current"
Capability assessment or maintenance	1. Disasters/pc
	2. (emergency response or emergency preparedness or emergency plan$ or emergency operation plan$ or disaster or major incident$ or incident plan$).ti,ab.
	3. 1 or 2
	4. (capability assessment or capability maintenance or gap analysis or needs assessment or drill or simulation or preparedness training).ti,ab.
	5. 3 and 4
	6. limit 5 to yr = "1990 -Current"
Recovery	1. Disasters/pc
	2. (emergency response or emergency preparedness or emergency plan$ or emergency operation plan$ or disaster or major incident$ or incident plan$).ti,ab.
	3. 1 or 2
	4. (significant event analysis or serious untoward incident$ or root cause analysis or debrief or organi?ational learning or rehabilitation).ti,ab.
	5. 3 and 4
	6. limit 5 to yr = "1990 -Current"
Communications/ informatics	1. Disasters/pc
	2. (emergency response or emergency preparedness or emergency plan$ or emergency operation plan$ or disaster or major incident$ or incident plan$).ti,ab.
	3. 1 or 2
	4. (communication$ or mass media or public relations or information system$ or information service$).ti,ab.
	5. 3 and 4
	6. limit 5 to yr = "1990 -Current"
Organisational behaviour	1. Disasters/pc
	2. (emergency response or emergency preparedness or emergency plan$ or emergency operation plan$ or disaster or major incident$ or incident plan$).ti,ab.
	3. 1 or 2
	4. (community engagement or community involvement or participatory involvement or participatory engagement or consumer participation or organi?ational behavio?r or health personnel or human resources).ti,ab.
	5. *"Attitude of Health Personnel"/
	6. *Interprofessional Relations/
	7. 4 or 5 or 6
	8. 3 and 7
	9. limit 8 to yr = "1990 -Current"

**Figure 1:**
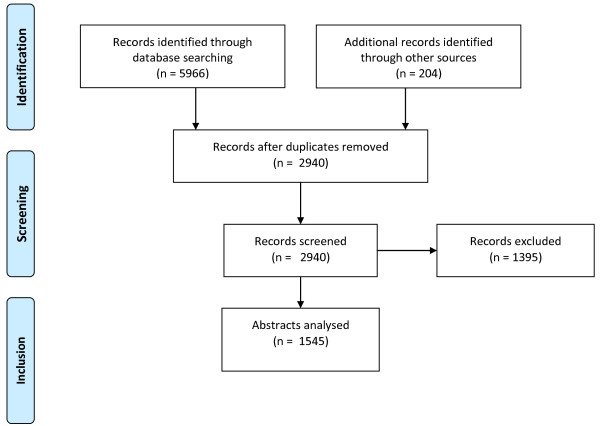
Limited PRISMA flow diagram.

Extracted titles and abstracts were screened by KC, AL, PG and AB. Each title or abstract was reviewed and deemed to be relevant, equivocal (subject matter suggestive of relevance to emergency planning and/or management), not relevant or containing inadequate information for coding (limited PRISMA chart figure 1). To operationalise the brief from the commissioners of the study to prioritise research relevant to UK health emergency planning we sought to include literature relating to comparable health services. We therefore excluded articles relating to non-health emergency planning, non-emergency planning, and non-UK legislative issues, and those from low- and middle-income countries unless they were likely to be generalisable to the UK. Two subsets of two hundred references were coded by a pair of researchers (either KC/PG or AL/AB) and kappa values for agreement calculated to examine consistency.

### Grey literature identification

We aimed to identify grey literature that included primary data or analysis that could be used to inform decision-making. Potential sources of grey literature were identified in advance by our expert advisory group and supplemented by the academic literature review and concurrent stakeholder interviews (reported elsewhere). Based on this, we searched databases at the Health Protection Agency, NHS Evidence, British Association of Immediate Care Schemes, Emergency Planning College and the Health and Safety Executive, and the websites of UK Department of Health Emergency Planning Division and UK Resilience. We also conducted an internet search using Google and Internet Archive for publicly accessible reports (public enquiries and coroner’s reports) into the following previously identified major incidents:

Aberfan slag heap slip

Hillsborough stadium disaster

Ibrox stadium incident

July 7 bombings

Kegworth (M1) aircraft crash

Ladbroke Grove rail

Lockerbie bombing

Manchester airport take-off crash

Marchioness pleasure boat sinking

Piper Alpha oil rig explosion

Potters Bar rail

Summerland fire disaster

Ufton Nervet rail crash

Zeebrugge ferry sinking

### Literature analysis and synthesis

**Figure 2:**
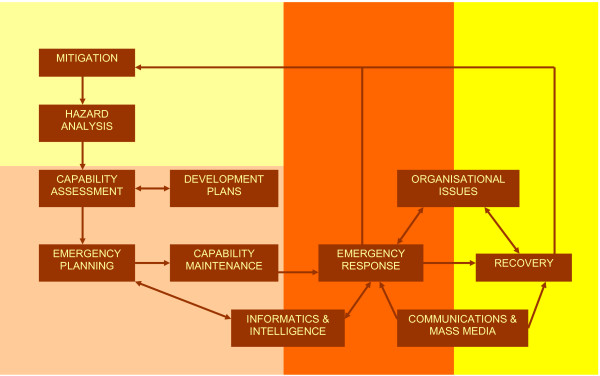
Adapted Emergency Management Cycle.

Where the title and abstract were considered to be relevant or equivocal we extracted further information relating to country of origin, type of publication and type of event discussed. Publications were coded on an Microsoft Excel spreadsheet using aggregative synthesis, a technique appropriate for exploring qualitative data where the concepts are secure, predefined and not contested[8]. We used a thematic framework developed a priori from the FEMA Emergency Management Cycle (figure 2)[9]. This framework is widely known and used within the emergency planning community, and covers the range of issues in the field using mutually exclusive concepts. Framework synthesis can be used in conjunction with either aggregative or interpretive reviews, has been shown to facilitate more rapid coding of the literature[10] and is, therefore, particularly suited to the objectives of a scoping review.

## Results

### Academic literature review

We reviewed 2940 titles and abstracts. Of these, 625 were coded by a pair of reviewers and with kappa values of 0.578 (AL/AB) and 0.740 (KC/PG). 1545 publications were felt to be relevant or equivocal. The country, type of publication and type of event are shown in Tables 2 and 3. For comparison, the number of events reported to the EM-DAT database at the Centre for Research on the Epidemiology of Disasters from 1990–2011 is also shown[11]. EM-DAT is a multi-source validated database of disasters where 10 or more people are killed, 100 or more affected, a state of emergency is declared or a call for international assistance is made. As we deliberately excluded much of the literature from low- and middle-income countries, percentages shown relate to EM-DAT records for high-income countries. This shows that the US and Canada are relatively over-represented in the literature, while continental Europe, Australasia and Japan are relatively under-represented.

**Table 2:** Country demographics of publications

	**Number of publications**	**%age publications**	**Number of EMDAT entries 1990–2011 n = 13378**	**%age EMDAT entries from high income countries (n = 2777)**
Country				
UK	47	3.0	86	3.1
US/Canada	701	45.4	755	27.2
Europe (not UK)	114	7.4	937	33.7
Japan	45	2.9	149	5.4
Australasia	26	1.7	359	12.9
Other high-income	51	3.3	491	17.7
Low/middle-income	178	11.5	10601	
Multiple	75	4.9		
Unspecified	308	19.9		

**Table 3:** Disaster and publication type

	**Number academic publications**	**%age academic publications**	**Number of grey documents**	**%age of grey documents**
Disaster type				
Natural eg earthquake	339	21.9	16	16.5
CBRN	135	8.7	44	45.4
Terrorism	119	7.7	8	8.2
Outbreak/epi/pandemic	89	5.8	7	7.2
Industrial	42	2.7	12	12.3
Transport	35	2.3	2	2.1
Generic	647	41.9	5	5.2
Multiple	84	5.4	3	3.1
Other	55	3.5		
Publication type				
Commentary/editorial	422		9	9.3
Event report	371		74	76.3
Expert guidance	122		4	4.1
Survey	118			
Educationalist	75		1	1
Modelling	73			
Narrative review	51		1	1
Systematic review	11			
RCT	2			
Other	300		8	8.2

Themes addressed in each publication are shown in Table 4. Table 5 shows the thematic analysis of publications in the event report, narrative review, systematic review, modelling, survey and RCT categories. These demonstrate a predominance of publications (over half) relating to emergency planning and emergency response, with relatively few addressing mitigation, with 219 of 362 event reports describing the emergency response.

**Table 4:** Thematic analysis of publications

**Theme**	**Number of academic publications**	%age of academic publications*	**Number of grey documents**	%age of grey documents*
Mitigation	50	3.1	18	18.6
Hazard analysis	124	7.8	27	27.8
Capability assessment	167	10.5		
Emergency planning	391	24.5	30	30.1
Capability maintenance	235	14.7	10	10.3
Emergency response	557	34.9	53	54.6
Recovery	177	11.1	27	27.8
Development plans	91	5.7		
Communications/mass media	156	9.8	35	36.1
Informatics and intelligence	183	11.5	26	26.8
Other organisational issues	183	11.5	35	36.1

*Total exceeds 100% due to multiple themes in individual publications.

**Table 5:** Thematic analysis by publication type

	**Event report (n = 362)**	**Narrative review (n = 53)**	**Systematic review (n = 11)**	**Modelling (n = 73)**	**Survey (n = 118)**	**RCT (n = 2)**
Mitigation	5	2		3	1	
Hazard analysis	7	3		29	8	
Capability assessment	17	6	2	9	45	1
Emergency planning	57	24	4	19	33	
Capability maintenance	34	6	1	6	36	1
Emergency response	219	19	6	14	22	1
Recovery	49	7	2	2	9	
Development plans	16	3	1		3	
Communications/ mass media	33	3		3	8	
Informatics/ intelligence	28	3		21	7	
Other organisational issues	32	8		1	9	

*Total in each column exceeds n due to multiple themes in individual publications.

### Grey literature review

192 documents were initially identified, of which 97 were relevant. Of these, 52 were published by the Health Protection Agency. Table 3 shows the type of publication and type of event. The preponderance of publications relating to CBRN probably reflects the accessibility of Health Protection Agency reports. As in the academic literature, the majority of documents addressed emergency response, although there was significant discussion of organisational issues and communications.

## Discussion

This scoping review demonstrates that the published literature relating to health emergency planning is disproportionately centred on North America; Australasia and Europe have produced surprisingly little academic literature given the number of reported incidents in these areas, although non-English publications from Europe were excluded from the scope of this review. The proliferation of emergency planning research in the US may represent the effect of widespread federal funding following the 9/11 attacks.

To our knowledge this represents the first attempt to scope the emergency planning literature relevant to a UK setting. This scoping review used a deliberately inclusive strategy and therefore our findings may over-estimate the extent of useable evidence. Equally, we did not assess the methodological quality of individual publications beyond their stated design. It is unclear whether the traditional hierarchy of evidence applied to clinical research (privileging meta-analyses and randomised controlled trials)[12] is appropriate in this field. Except in very specific areas (for example brief psychological interventions for survivors) randomised controlled trial designs are not practical. Narrative synthesis of observational studies may be possible[13] but is currently hampered by the unevenness of collection. Repeated calls have been made to standardise major incident reporting and suggestions exist for the format this should take[14,15]. Even though multiple event reports exist there is little indication that this potential evidence base has been fully exploited through synthesis. We found it difficult to identify a repository of emergency planning literature that was easily and openly accessible. The two main repositories were the HPA’s Chemical Incidents and Poisons Report database and the Emergency Planning College library. Access to the Emergency Planning College library at Easingwold is controlled and their collection is not open to the public.

We have identified a substantial amount of literature relating to emergency response. However, the validity and generalisability of the data is unclear, as is its amenability to synthesis and how this should inform policy and practice. We have also exposed the limited evidence base available to assist emergency planners and policymakers in the areas of mitigation and recovery. The development of an evidence base for emergency planning needs to take into account practical considerations regarding what type of evidence is most likely to be influential, as well as theoretical considerations of what constitutes valid evidence. The type and structure of evidence that would be of most value of emergency planners and policymakers has yet to be identified. Further research into stakeholder perceptions will be required to determine this.

## Conclusions

A significant body of emergency planning literature exists. Nevertheless its usefulness to UK planners is constrained because it is dominated geographically by the US, thematically by emergency response topics and methodologically by event reports.

## Competing interests

The authors declare that they have no competing interests.

## Authors’ contributions

SG, AL, KC and AB conceived the study. AB and HBW developed and carried out the academic literature search. AB, AL, KC and PG coded the academic literature. PG carried out the grey literature search which was coded by KC and AL. All authors had full access to the data and have approved the final manuscript. KC is the guarantor.

## Ethics approval

Ethical approval was not required.

## Pre-publication history

The pre-publication history for this paper can be accessed here:

http://www.biomedcentral.com/1471-2458/12/542/prepub
